# Effect of CFTR modulator therapy with elexacaftor/tezacaftor/ivacaftor on pulmonary ventilation derived by 3D phase-resolved functional lung MRI in cystic fibrosis patients

**DOI:** 10.1007/s00330-023-09912-6

**Published:** 2023-08-07

**Authors:** Filip Klimeš, Andreas Voskrebenzev, Marcel Gutberlet, Milan Speth, Robert Grimm, Martha Dohna, Gesine Hansen, Frank Wacker, Diane Miriam Renz, Anna-Maria Dittrich, Jens Vogel-Claussen

**Affiliations:** 1 Institute of Diagnostic and Interventional Radiology, Hannover Medical School, Hannover, Germany; 2 Biomedical Research in Endstage and Obstructive Lung Disease Hannover (BREATH), German Center for Lung Research (DZL), Hannover, Germany; 3 MR Application Predevelopment, Siemens Healthcare GmbH, Erlangen, Germany; 4 Department for Pediatric Pneumology, Allergology and Neonatology, Hannover Medical School, Hannover, Germany

**Keywords:** Cystic fibrosis, Magnetic resonance imaging, Ventilation

## Abstract

**Objectives:**

To investigate whether 3D phase-resolved functional lung (PREFUL)-MRI parameters are suitable to measure response to elexacaftor/tezacaftor/ivacaftor (ETI) therapy and their association with clinical outcomes in cystic fibrosis (CF) patients.

**Methods:**

Twenty-three patients with CF (mean age: 21; age range: 14–46) underwent MRI examination at baseline and 8–16 weeks after initiation of ETI. Morphological and 3D PREFUL scans assessed pulmonary ventilation. Morphological images were evaluated using a semi-quantitative scoring system, and 3D PREFUL scans were evaluated by ventilation defect percentage (VDP) values derived from regional ventilation (RVent) and cross-correlation maps. Improved ventilation volume (IVV) normalized to body surface area (BSA) between baseline and post-treatment visit was computed. Forced expiratory volume in 1 second (FEV_1_) and mid-expiratory flow at 25% of forced vital capacity (MEF25), as well as lung clearance index (LCI), were assessed. Treatment effects were analyzed using paired Wilcoxon signed-rank tests. Treatment changes and post-treatment agreement between 3D PREFUL and clinical parameters were evaluated by Spearman’s correlation.

**Results:**

After ETI therapy, all 3D PREFUL ventilation markers (all *p* < 0.0056) improved significantly, except for the mean RVent parameter. The BSA normalized IVV_RVent_ was significantly correlated to relative treatment changes of MEF25 and mucus plugging score (all |*r*| > 0.48, all *p* < 0.0219). In post-treatment analyses, 3D PREFUL VDP values significantly correlated with spirometry, LCI, MRI global, morphology, and perfusion scores (all |*r*| > 0.44, all *p* < 0.0348).

**Conclusions:**

3D PREFUL MRI is a very promising tool to monitor CFTR modulator–induced regional dynamic ventilation changes in CF patients.

**Clinical relevance statement:**

3D PREFUL MRI is sensitive to monitor CFTR modulator–induced regional ventilation changes in CF patients. Improved ventilation volume correlates with the relative change of mucus plugging, suggesting that reduced endobronchial mucus is predominantly responsible for regional ventilation improvement.

**Key Points:**

*• 3D PREFUL MRI–derived ventilation maps show significantly reduced ventilation defects in CF patients after ETI therapy.*

*• Significant post-treatment correlations of 3D PREFUL ventilation measures especially with LCI, FEV*_*1*_ *%pred, and global MRI score suggest that 3D PREFUL MRI is sensitive to measure improved regional ventilation of the lung parenchyma due to reduced inflammation induced by ETI therapy in CF patients.*

*• 3D PREFUL MRI–derived improved ventilation volume (IVV) correlated with MRI mucus plugging score changes suggesting that reduced endobronchial mucus is predominantly responsible for regional ventilation improvement 8–16 weeks after ETI therapy.*

## Introduction

Cystic fibrosis (CF) is an autosomal recessive genetic disorder of the cystic fibrosis transmembrane conductance regulator (CFTR) protein, which reduces chloride and sodium ion transport across cell membrane of epithelial barriers. Consequently, the airways are filled with thick mucus that restricts breathing. CF occurs in approximately 1 in 3000–4000 newborns among Caucasians [1]. Thus, early diagnosis and monitoring of therapy is of significant interest. Recently, a new CFTR modulator combination medication, composed of a chloride channel potentiator (ivacaftor) and two CFTR correctors (elexacaftor and tezacaftor), has been introduced to treat CF. The first studies with this triple-combination therapy, henceforth abbreviated ETI, demonstrated significant improvements in clinical outcomes in CF patients [2–4].

The ventilation outcomes in CF are routinely measured using spirometry and nitrogen multiple-breath washout (N_2_-MBW). Both techniques estimate global pulmonary ventilation parameters. However, they cannot assess regional information. Chest CT allows for regional monitoring of early lung disease [5]. In contrast to CT, MRI offers radiation-free imaging and therefore the possibility for follow-up measurements without accumulating radiation exposure.

In the last 15 years, several MRI techniques have been developed to assess regional ventilation during free breathing and without the usage of contrast-agent [6–10]. Among them, 3D phase-resolved functional lung (PREFUL) MRI enables quantitative assessment of pulmonary ventilation during free breathing on a regional level of the total lung volume [11]. In contrast to 2D alternatives, 3D techniques [11–13] offer whole lunge coverage (better spatial resolution and likely higher sensitivity for hypoventilated regions) and account for through-plane motion, which potentially improve the quality of functional ventilation maps. 3D PREFUL ventilation parameters have been shown to correlate well with spirometry measurements and showed a good interscan repeatability in a study cohort consisting of healthy volunteers and chronic obstructive pulmonary disease patients [14]. Recently, ETI therapy has been shown to improve global clinical ventilation parameters and semi-quantitative morphologic MRI scoring in CF patients [15]. However, it is unknown whether 3D PREFUL ventilation parameters are sensitive to measure treatment changes in patients with CF.

The objective of this study was to investigate if the ventilation parameters derived by 3D PREFUL are suitable to measure response to ETI therapy and their association with improvements in clinical outcome measures in CF patients.

## Materials and methods

### Study participants

A total of 23 CF patients (13 female, mean age *±* standard deviation (SD), 21.0 *±* 9.3 years, mean FEV_1_ % pred. at baseline 87.8%) were recruited in this prospective observational single-center study. The local ethics committee of Hannover Medical School (8922_B0_S_2020) approved the study and informed written consent was obtained prior to the examination from each study participant and/or their legal guardians. Participants were included when they (1) were older than 12 years, (2) were compound-heterozygous for F508del and a minimal function mutation or homozygous for F508del, and (3) had no prior exposure to ETI. The following exclusion criteria were applied: (1) pulmonary exacerbation at baseline or follow-up, (2) history of solid organ transplantation, (3) pregnancy, (4) claustrophobia, (5) metallic implantations incompatible with MR imaging. Morphological and functional 3D PREFUL MRI, PFT, and N_2_-MBW were evaluated at baseline and 8–16 weeks after initiation of ETI therapy. The morphological MRI images, spirometry data, and LCI values of the included CF patients have been reported in a previous study [15].

### MR protocol

MR examinations were performed on a 1.5-T scanner (MAGNETOM Avanto; Siemens Healthcare). The MR protocol consisted of the following sequences [15]: (1) T2-weighted half-Fourier acquisition single-shot turbo spin echo (HASTE) sequences in axial and coronal orientation, acquired by breath-hold maneuvers; (2) T2-weighted turbo spin echo sequences in PROPELLER technique (BLADE), acquired in axial and coronal orientation by using respiratory navigator triggering; (3) T1-weighted volumetric interpolated breath-hold examination (VIBE) sequences, acquired in axial and coronal orientation. (4) 2D non-contrast-enhanced PREFUL technique to assess lung perfusion and 3D PREFUL technique to asses lung ventilation.

For 3D PREFUL, an 8-min measurement [11] was used to assess regional lung ventilation of the whole lung. The following sequence parameters were used for the 3D PREFUL: echo time (TE) 0.81 ms, repetition time (TR) 1.9 ms, flip angle 3.5°, field of view (FOV) 45 × 45 cm^2^, matrix size 112 × 112 interpolated to 224 × 224, 52–60 partitions, 6/8 partial Fourier along the partition dimension, pixel bandwidth 1500 Hz/pixel, slice thickness 4 mm interpolated to 2 mm.

### MR image analysis

Morphological MR images were evaluated based on a well-established semi-quantitative scoring system [16]. Briefly, the global MRI score consists of morphology score and perfusion score. The morphology score is divided into the following subscores: bronchial wall thickening/bronchiectasis, mucus plugging, abscesses/sacculations, consolidations, and special findings, i.e., pleural affection. The perfusion scores were derived by the subtraction of the baseline images (contrast-free) from the images with maximal contrast in the lung tissue [16]. All subscores including the perfusion score are evaluated for each lobe with values of (a) 0 (no abnormality), (b) 1 (< 50% of the lobe affected), and (c) 2 (≥ 50% of the lobe affected). Considering lingula as a sixth lobe, the global MRI score ranges from 0 (normal lung) to 72 (maximally affected lung).

The dynamically acquired 3D PREFUL data were reconstructed to ~40 breathing phases and each phase was registered towards end-inspiration image [17]. Using 3D PREFUL registered images, ventilation defect maps derived from static regional ventilation (RVent) [18] and dynamic cross-correlation (CC) [9] maps based on previously published thresholds were calculated [14]. CC maps assess the dynamics of ventilation using regional flow-volume loops during tidal breathing. Furthermore, ventilation defect percentage (VDP) percent values were computed from both ventilation defect maps as the number of voxels with ventilation below the threshold divided by the total number of voxels of the segmented lung volume. To analyze the treatment effect on a regional level, morphological images of the second visit were co-registered to the morphological end-inspiratory image of the first visit. The treatment response maps (TRMs) were then calculated by subtracting the ventilation defect map after therapy from the baseline ventilation defect maps. Post-treatment improved ventilation volume (IVV_RVent_ and IVV_CC_) was calculated as the number of positively changed voxels in TRMs (absolute amount of ventilation defect voxels at baseline measurement which resolved after ETI therapy in the follow-up measurement) multiplied by lung volume in milliliters. Both volumes were normalized to body surface area (BSA) at baseline. For image analysis, the lung parenchyma of the 3D PREFUL baseline end-inspiratory reconstructed image was automatically segmented using a 3D convolutional neuronal network (CNN) and manually corrected if needed. The details of the 3D CNN network were described previously [17].

### Lung function testing

Spirometry outcomes (forced expiratory volume in 1 second (FEV_1_) and mid-expiratory flow at 25% of forced vital capacity (MEF25)) were obtained according to American Thoracic Society (ATS)/European Respiratory Society (ERS) guidelines [19] with global lung function initiative (GLI) reference values [20]. Lung clearance index (LCI) was assessed using the N_2_-MBW technique according to ATS/ERS guidelines [21].

### Statistical analysis

For the 3D PREFUL regional parameters of RVent and CC, mean values and SD values inside lung parenchyma were computed. Further ventilation impairment was assessed using 3D PREFUL by a total lung VDP and post-treatment IVV values in milliliters.

The treatment effect on all derived MRI and clinical outcome parameters was analyzed using paired Wilcoxon signed-rank tests.

Treatment changes and post-treatment agreement between 3D PREFUL and clinical parameters were evaluated using Spearman’s correlation (*r*) analyses.

For all comparisons, *p* values < 0.05 were considered statistically significant. Statistical analyses were performed using JMP Pro 16 (SAS Institute) and MATLAB 2020b (MathWorks).

## Results

### Study participants

MRI scans and PFT measurements were successfully performed in all 23 CF patients. Two out of 23 CF patients did not comply with N_2_-MBW measurement at the follow-up visit. Due to coronavirus pandemic-related restrictions, the follow-up measurement of seven out of 23 CF patients (30%) had to be postponed up to 19 weeks with one patient exception (28 weeks).

### Effects of ETI therapy on MRI and associated lung function data

**Fig. 1:**
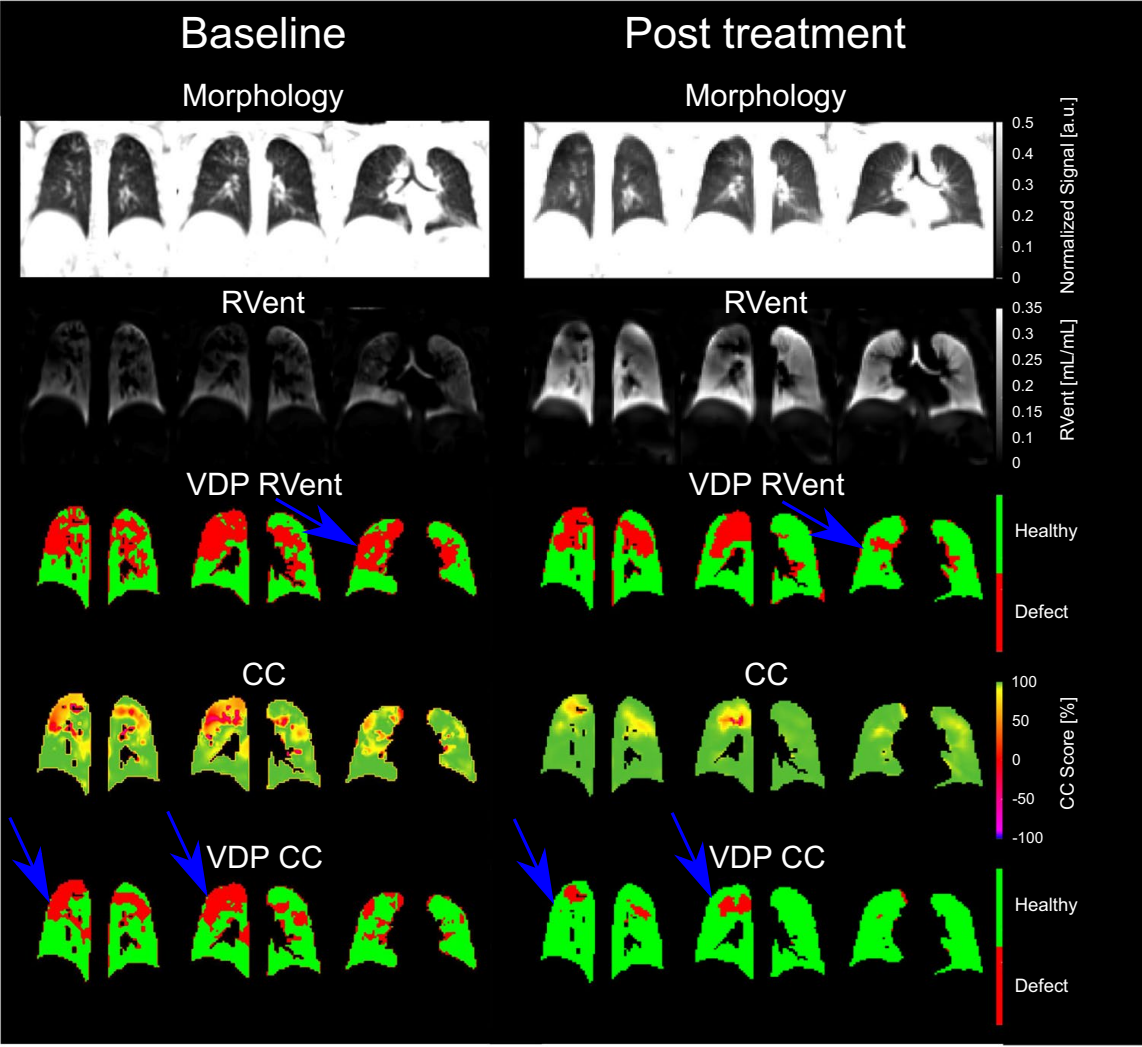
Exemplary ventilation marker maps of baseline (left) and post-treatment (right) 3D PREFUL measurements of 42-year-old female CF patient. VDP_RVent_ decreased from 31.8% (baseline) to 19.9% (post-treatment). VDP_CC_ decreased from 18.0% (baseline) to 3.6% (post-treatment). FEV_1_ % pred. baseline: 94%. FEV_1_ % pred. post-treatment: 112%. MEF25% pred. baseline: 31%. MEF25% pred. post-treatment: 68%. LCI baseline: 10.48, LCI post-treatment: 9.39. Global score baseline: 25. Global score post-treatment: 18. Morphology score baseline: 20. Morphology score post-treatment: 13. The blue arrows show the resolved ventilation after therapy with ETI

**Fig. 2:**
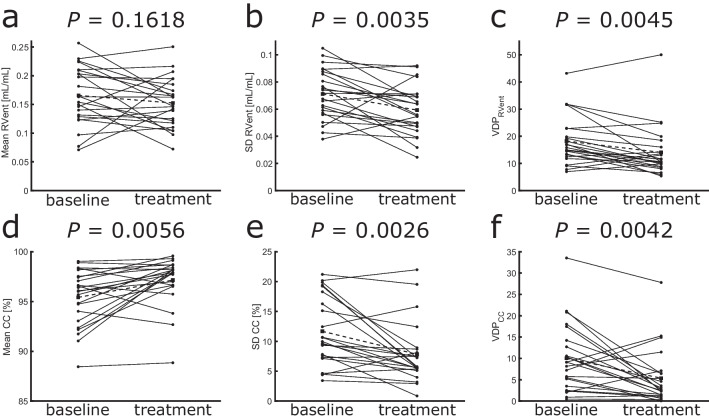
Effect of ETI treatment on ventilation parameters derived by 3D PREFUL. Paired measurements of mean RVent (**a**), SD RVent (**b**), VDP_RVent_ (**c**), mean CC (**d**), SD CC (**e**), and VDP_CC_ (**f**) at baseline and after initiation of ETI therapy. *p* < 0.05 were considered statistically significant. The black dashed line shows the average change between baseline and post-treatment in each ventilation parameters

Therapy with ETI significantly improved all ventilation markers derived by 3D PREFUL MRI (range of improvement 1.9–28.1%, all *p* < 0.0056, Table 1) as well as MRI global, morphology, and perfusion scores (range of improvement 9.5–31.9%, all *p* < 0.02, Table 1), except for the 3D PREFUL–derived mean RVent parameter (improvement 0.6%, *p* = 0.16). As reported previously [15], FEV_1_, MEF25, and LCI significantly improved after therapy (range of improvement 22.4–74.7%, all *p* < 0.0001, Table 1). Representative 3D PREFUL ventilation parameters at baseline and post-ETI treatment initiation are depicted in Fig. 1. Treatment effects on 3D PREFUL ventilation parameters are depicted in Fig. 2.

**Table 1:**
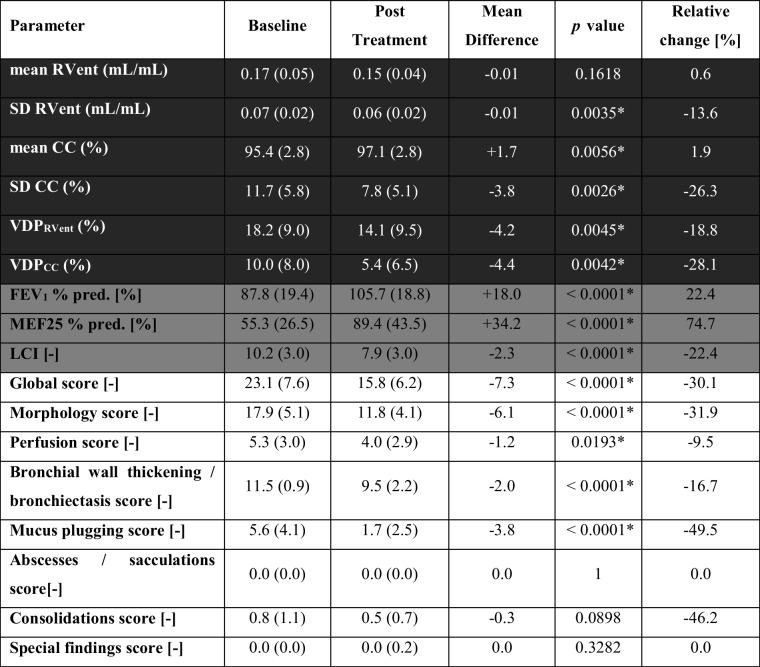
Analysis of 3D PREFUL, spirometry, N_2_-MBW, and morphological MRI parameters in response to ETI therapy. Baseline and post-treatment ventilation values are expressed as average with standard deviation in brackets. Significant results are marked with *

### Comparison of treatment changes between 3D PREFUL and lung function data

**Fig. 3:**
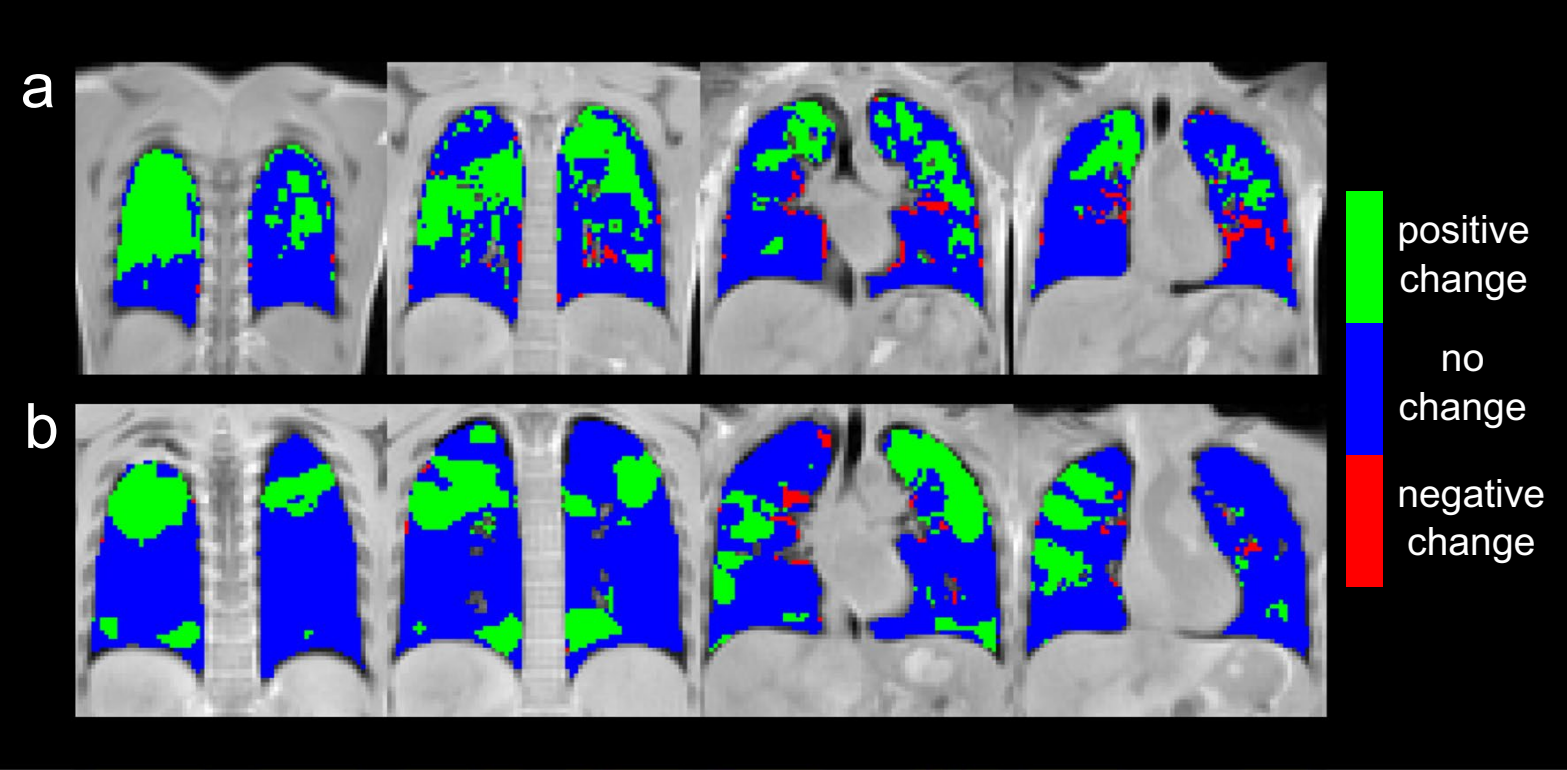
In **a**, exemplary treatment response maps for a 26-year-old female derived from ventilation defect maps of RVent parameter. The positive change of 24.2% (IVV_RVent_/BSA = 442 mL/m^2^) compared to the negative change of 3.1%, resulted in an improvement of 21.1%. In **b**, examples of coronal treatment response maps for a 19-year-old male derived from ventilation defect maps of CC parameter are shown. Subtracting the negative change of 1.3% from the positive change of 16.1% (IVV_CC_/BSA = 358 mL/m^2^) results in an improvement of 14.8%

There was no significant correlation between absolute and relative treatment changes of 3D PREFUL MRI ventilation parameters (mean RVent, SD RVent, mean CC, SD CC, VDP_RVent_, and VDP_CC_) with FEV_1_, MEF25, LCI, and MRI scores (*p* > 0.05), except relative change of SD RVent parameter, which was significantly correlated with relative change of LCI (*r* = 0.48, *p* = 0.0275). Figure 3 shows regional TRMs derived by 3D PREFUL for two study participants. Looking only at positive changes in TRMs, the mean improved ventilation volume normalized to BSA was 147 (SD = 94) and 129 (SD = 114) mL/m^2^ for RVent (IVV_RVent_) and CC (IVV_CC_) parameters, respectively. The IVV_RVent_ normalized to BSA was significantly correlated with relative treatment changes of MEF25 and mucus plugging MRI score (all |*r*| > 0.48, all *p* < 0.219, Table 2).

**Table 2:** Spearman’s correlation analysis of improved ventilation volumes (IVV_RVent_ normalized to body surface area (BSA) and IVV_CC_ normalized to BSA, respectively) derived by 3D PREFUL and % change of clinical parameters. Statistically significant correlations are marked with * (*p* value < 0.05)

Parameter	IVV_RVent_/BSA		IVV_CC_/BSA	
	*r*	*p* value	*r*	*p* value
*FEV*_*1*_* % pred.*	*0.28*	*0.2046*	*0.33*	*0.1217*
*MEF25 % pred.*	*0.48*	0.0219*	*0.27*	*0.2115*
*LCI*	*0.02*	*0.9199*	*0.09*	*0.7076*
MRI global score	−0.02	0.9393	−0.20	0.3532
MRI morphology score	−0.00	0.9946	−0.17	0.4361
MRI perfusion score	−0.07	0.7470	0.08	0.7113
MRI bronchial wall thickening/bronchiectasis score	0.08	0.7209	−0.06	0.7933
MRI mucus plugging score	−0.49	0.0181*	−0.22	0.3242
MRI consolidation score	−0.13	0.5447	−0.18	0.4220

### Comparison of post-treatment values between 3D PREFUL and lung function outcome parameters

**Fig. 4:**
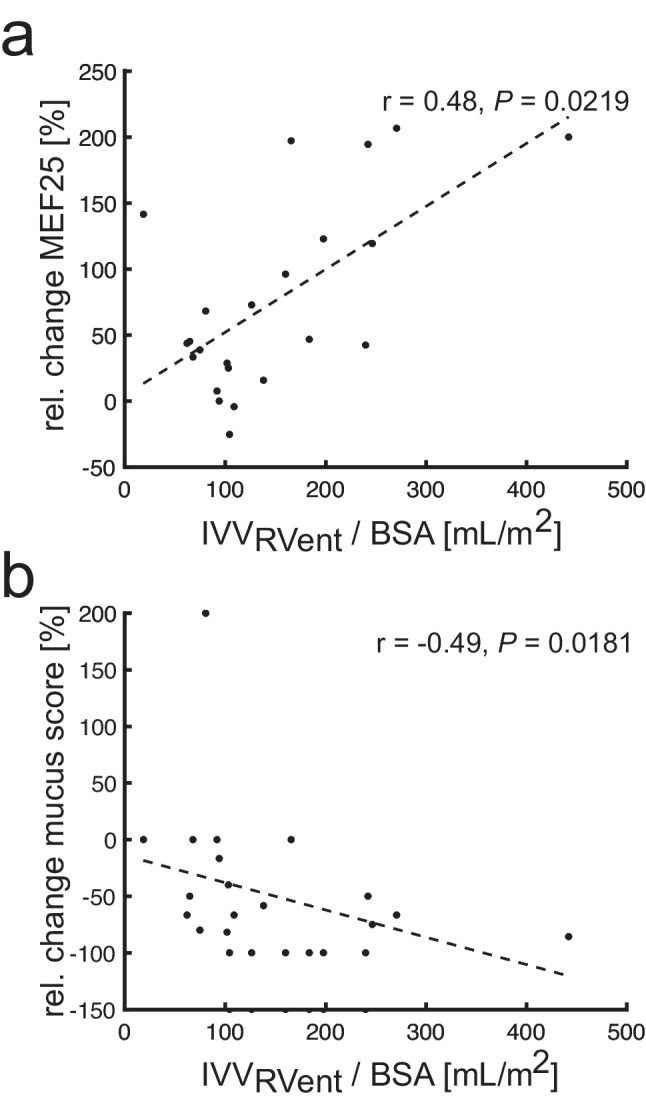
Spearman’s correlation analysis of improved ventilation volume IVV_RVent_ normalized to BSA with relative change of MEF25 % pred. (top row (**a**)) and relative change of mucus plugging score (bottom row (**b**)). Black circles represent the patients (*n* = 23) and dashed black lines represent simple linear regression, *r* = Spearman’s correlation coefficient

In post-treatment analyses, 3D PREFUL–derived VDP values significantly correlated with spirometry, LCI, global score, morphology score, and perfusion score (all |*r*| > 0.44, all *p* < 0.0348, Table 3, Fig. 4), except for correlation of VDP_CC_ with global and morphology score. CC values derived by 3D PREFUL correlated significantly with FEV_1_, MEF25, and LCI (all |*r*| > 0.49, all *p* < 0.0253, Table 3).

**Table 3:** Post-treatment Spearman’s correlation analysis of 3D PREFUL ventilation parameters and lung function parameters (spirometry, N_2_-MBW) and morphological MRI parameters assessed by semi-quantitative scoring system. Statistically significant correlations are marked with * (*p* value < 0.05)

Parameter	Mean RVent		SD RVent		Mean CC		SD CC		VDP_RVent_		VDP_CC_	
	Spearman's r	*p* value	Spearman’s *r*	*p* value	Spearman’s *r*	*p* value	Spearman’s *r*	*p* value	Spearman’s *r*	*p* value	Spearman’s *r*	*p* value
*FEV*_*1*_* % pred.*	*0.43*	0.0390*	*−0.07*	*0.7417*	*0.63*	0.0014*	*−0.60*	0.0024*	*−0.58*	0.0040*	*−0.71*	0.0001*
*MEF25 % pred.*	*0.17*	*0.4283*	*−0.27*	*0.2171*	*0.62*	0.0015*	*−0.52*	0.0109*	*−0.58*	0.0037*	*−0.66*	0.0007*
*LCI*	*−0.32*	*0.1597*	*0.15*	*0.5217*	*−0.49*	0.0253*	*0.35*	*0.1190*	*0.50*	0.0217*	*0.55*	0.0093*
MRI global score	−0.14	0.5097	0.24	0.2629	−0.30	0.1599	0.29	0.1724	0.60	0.0024*	0.39	0.0690
MRI morphology score	−0.35	0.0990	0.02	0.9262	−0.26	0.2342	0.20	0.3543	0.54	0.0076*	0.34	0.1141
MRI perfusion score	0.04	0.8654	0.45	0.0315*	−0.36	0.0905	0.41	0.0546	0.62	0.0015*	0.44	0.0348*
MRI bronchial wall thickening/bronchiectasis score	−0.51	0.0130*	−0.18	0.4225	−0.23	0.2997	0.10	0.6613	0.30	0.1648	0.30	0.1663
MRI mucus plugging score	−0.03	0.8767	0.21	0.3308	−0.18	0.4247	0.18	0.4041	0.50	0.0158*	0.25	0.2588
MRI consolidation score	0.00	0.9836	0.07	0.7501	−0.10	0.6471	0.15	0.4926	0.20	0.3590	0.10	0.6471

## Discussion

This study assessed the effects of ETI therapy on pulmonary ventilation parameters derived by 3D PREFUL MRI in patients with CF. 3D PREFUL–derived ventilation parameters showed significantly reduced ventilation defects after initiation of ETI therapy and the improved ventilation volume measure was significantly correlated to the relative change in the morphological parameter mucus plugging and MEF25.

To our best knowledge, this is the first study to determine the effects of ETI therapy on pulmonary ventilation function using patient-friendly non-contrast-enhanced 3D MR acquisition during tidal breathing. As reported previously [15, 22], the improvements after initiation of ETI were also found for clinical outcome parameters, including spirometry and N_2_-MBW ventilation parameters, as well as for semi-quantitative MRI scores. Improved ventilation parameters after ETI therapy using dynamic perfluorinated gas ^19^F MR imaging have been previously reported in one study including 8 CF patients [23]. The authors revealed an absolute change difference of VDP values of −2.7% (relative change −33%), which is slightly lower compared to our results of −4.2% VDP_RVvent_ (relative change −23%) and −4.4% VDP_CC_ (relative change −46%). A direct comparison of our VDP values with mentioned ^19^F imaging VDP results is challenging due to different study design, signal generation differences, breathing conditions (free tidal breathing for 3D PREFUL vs. inspiration breath-hold for ^19^F), spatial resolution (4 x 4 x 4 mm^3^ for 3D PREFUL compared to 6.25 × 6.25 × 15 mm^3^ for ^19^F), and scanner field strengths (3D PREFUL at 1.5T vs. ^19^F at 3T).

As 3D PREFUL covers the whole lung volume, it is feasible to register pre- and post-treatment acquisitions and calculate treatment response ventilation maps, which enable a detailed longitudinal regional treatment change analysis [24]. Regarding relative treatment change, we found significant correlations of 3D PREFUL MRI–derived improved ventilation volume (IVV_RVent_ normalized to BSA) with relative change of MEF25 and the mucus plugging MRI score. These findings suggest that reduced endobronchial mucus is predominantly responsible for regional ventilation improvement 8–16 weeks after initiation of ETI therapy. The positive changes in MRI-derived mucus plugging and wall thickening/bronchiectasis score have been shown to be associated with the improvement of CFTR function, which might reflect the resolution of inflammation in CF patients [15]. De Vuyst et al [25] demonstrated that the ETI therapy reduces airway inflammation in CF. Although 3D PREFUL MRI is not able to assess inflammation directly, the previous studies support the hypothesis that the improved regional ventilation derived by 3D PREFUL is related to the reduction in inflammation induced by ETI therapy. Also, the correlation of the regional ventilation changes with the relative change of MEF25 may indicate that 3D PREFUL MRI is sensitive to airflow changes in the small airways. This appears reasonable as 3D PREFUL MRI measures regional ventilation in the lung parenchyma. However, this finding warrants further investigation in future studies.

Absolute and relative changes of 3D PREFUL parameters were not directly correlated to changes in FEV_1_, LCI, and MRI scoring system. The missing correlations might be explained by the relatively small sample size leading to the low statistical power of our analysis. Also, this finding may highlight the complementary value of 3D PREFUL MRI and clinically established measures such as FEV_1_ and LCI.

Examining the relationship between 3D PREFUL parameters and other ventilation measures, significant correlations with post-treatment initiation FEV_1_, MEF25, LCI, global MRI score, morphology MRI score, perfusion MRI score, bronchial wall thickening/bronchiectasis MRI score, and mucus plugging score. The highest correlations were observed for dynamic cross-correlation parameter (CC and VDP_CC_), which uses the information of the whole respiratory cycle, with spirometric measures. This finding suggests increased treatment response sensitivity of CC when compared to static RVent parameters derived by 3D PREFUL. Nonetheless, the significant post-treatment correlations of 3D PREFUL ventilation measures especially with FEV_1_, LCI, and global MRI score demonstrate that 3D PREFUL MRI is sensitive to measure improved regional ventilation of the lung parenchyma due to reduced inflammation and endobronchial mucus in response to ETI therapy in CF patients. In addition, the improved perfusion score in the presented cohort may also be indicative of resolving inflammatory changes in the lung parenchyma post-ETI treatment.

In addition to the small sample size, we acknowledge further limitations. Firstly, the ventilation parameters derived by PREFUL (or other Fourier-decomposition-based methods) are considered indirect measures for ventilation. The key assumption of these methods is that the signal intensity variations of the lung parenchyma are induced by different lung volumes [6]. This idea has been validated for 2D PREFUL with direct ^19^F MRI and ^129^Xe ventilation measurement [26, 27] but not (yet) for 3D methods. Secondly, the repeatability of 3D PREFUL–derived dynamic parameters in CF patients has not been examined yet. Recent repeatability results in chronic obstructive pulmonary disease patients and stable CF patients showed no significant bias between repeated measurements of 3D and 2D PREFUL, respectively [14, 28]. This supports our results that the differences in 3D PREFUL parameters between baseline and post-treatment acquisition are a true response to ETI therapy. Additional useful information might be provided by assessment of improvement in ventilation on the lobar level, however was beyond the scope of this work. Future multicenter validation studies are necessary to investigate if 3D PREFUL MRI regional ventilation assessment may add value to current clinical standard techniques such as spirometry, N_2_-MBW, semi-quantitative thoracic MRI scores, or gas-based MRI techniques.

## Conclusions

In summary, our study demonstrates that ETI therapy leads to improvement in lung ventilation determined by 3D PREFUL MRI in CF patients. Our data suggest that endobronchial mucus reduction is mainly responsible for regional ventilation improvement 8–16 weeks after initiation of ETI therapy.

## Abbreviations

ATS: American Thoracic Society
BLADE: Periodically rotated overlapping parallel lines with enhanced reconstruction (PROPELLER) in Siemens Healthcare MRI systems
BSA: Body surface area (m^2^)
CC: Cross-correlation (%)
CF: Cystic fibrosis
CFTR: Cystic fibrosis transmembrane conductance regulator
CNN: Convolutional neuronal network
ERS: European Respiratory Society
FEV_1_: Forced expiratory volume in 1 s (% predicted value)
FOV: Field of view (cm^2^)
GLI: Global lung function initiative
HASTE: Half-Fourier acquisition single-shot turbo spin echo
IVV_CC_: Improved ventilation volume derived from TRM_CC_ (mL)
IVV_RVent_: Improved ventilation volume derived from TRM_RVent_ (mL)
LCI: Lung clearance index
MEF25: Mid-expiratory flow at 25% of forced vital capacity (% predicted value)
N_2_-MBW: Nitrogen multiple-breath washout
PFT: Pulmonary functional testing
PREFUL: Phase-resolved functional lung
RVent: Regional ventilation (mL/mL)
TE: Echo time (ms)
TR: Repetition time (ms)
TRM_CC_: Treatment response map based on VDP_CC_
TRM_RVent_: Treatment response map based on VDP_RVent_
VDP_CC_: Ventilation defect percentage value based on CC (%)
VDP_RVent_: Ventilation defect percentage value based on RVent (%)
VIBE: Volumetric interpolated breath-hold examination

## Declarations

### Guarantor

The scientific guarantor of this publication is Jens Vogel-Claussen.

### Statistics and biometry

No complex statistical methods were necessary for this paper.

### Study subjects or cohorts overlap

Some study subjects or cohorts have been previously reported in Graeber et al (2022) American Journal of Respiratory and Critical Care Medicine, https://doi.org/10.1164/rccm.202110-2249OC.

### Methodology

- prospective
- observational
- performed at one institution
